# Diaphragm pacing and independent breathing in individuals with severe Pompe disease

**DOI:** 10.3389/fresc.2023.1184031

**Published:** 2023-07-31

**Authors:** Cristina Liberati, Barry J. Byrne, David D. Fuller, Chasen Croft, Teresa Pitts, Jessica Ehrbar, Carmen Leon-Astudillo, Barbara K. Smith

**Affiliations:** ^1^ Department of Pediatrics, Boston Children’s Hospital, Boston, MA, United States; ^2^ Department of Pediatrics, University of Florida, Gainesville, FL, United States; ^3^ Department of Physical Therapy, University of Florida, Gainesville, FL, United States; ^4^ Breathing Research and Therapeutics (BREATHE) Center, University of Florida, Gainesville, FL, United States; ^5^ Department of Surgery, University of Florida, Gainesville, FL, United States; ^6^ Department of Speech, Language and Hearing Sciences, University of Missouri, Columbia, MO, United States; ^7^ Dalton Cardiovascular Center Investigator, University of Missouri, Columbia, MO, United States

**Keywords:** Pompe disease, diaphragm, mechanical ventilation, breathing, phrenic stimulation

## Abstract

**Introduction:**

Pompe disease is an inherited disease characterized by a deficit in acid-α-glucosidase (GAA), an enzyme which degrades lysosomal glycogen. The phrenic-diaphragm motor system is affected preferentially, and respiratory failure often occurs despite GAA enzyme replacement therapy. We hypothesized that the continued use of diaphragm pacing (DP) might improve ventilator-dependent subjects' respiratory outcomes and increase ventilator-free time tolerance.

**Methods:**

Six patients (3 pediatric) underwent clinical DP implantation and started diaphragm conditioning, which involved progressively longer periods of daily, low intensity stimulation. Longitudinal respiratory breathing pattern, diaphragm electromyography, and pulmonary function tests were completed when possible, to assess feasibility of use, as well as diaphragm and ventilatory responses to conditioning.

**Results:**

All subjects were eventually able to undergo full-time conditioning via DP and increase their maximal tolerated time off-ventilator, when compared to pre-implant function. Over time, 3 of 6 subjects also demonstrated increased or stable minute ventilation throughout the day, without positive-pressure ventilation assistance.

**Discussion:**

Respiratory insufficiency is one of the main causes of death in patients with Pompe disease. Our results indicate that DP in Pompe disease was feasible, led to few adverse events and stabilized breathing for up to 7 years.

## 1. Introduction

Pompe disease is a rare autosomal recessive disorder caused by a mutation in the gene that encodes lysosomal enzyme acid-α-glucosidase (GAA). A deficiency or absence of GAA leads to glycogen accumulation in multiple organ systems, including cardiac, striated, and smooth muscles, visceral organs, and neuronal tissue (1–4). The accumulation of glycogen triggers a chain of biochemical and metabolic events that result in diffuse cell death in the involved tissues (1, 5, 6). The consequent impairment of the neurologic, neuromuscular and cardiovascular system leads to the typical features of the disease: developmental delay, loss of balance, progressive general hypotonia and muscular weakness, respiratory insufficiency, cardiomegaly, cardiorespiratory failure and, eventually, death (7–10).

Based on age and severity of onset, the patients can be classified as early-onset (infantile, EOPD) or later-onset (juvenile or adult, LOPD) types. The early-onset form is a severe life-threatening condition characterized by profound hypotonia and weakness that, when untreated, inevitably leads to cardiorespiratory failure and death of affected patients (11). In contrast, the later-onset forms are notable for a slow, progressive deterioration of strength and ventilatory function, without significant cardiac involvement. Ventilatory muscle weakness and hypoventilation are predominant and common features with disease progression of both early- and later-onset Pompe disease (12–16). Commercially-available enzyme replacement therapy (ERT) infusions supplement innate GAA production, to preserve walking and decelerate respiratory declines (17–20). Respiratory failure and ventilator dependence are inevitable outcomes. Diaphragm functional impairment is well documented in Pompe disease (15, 16), but an accumulation of evidence indicates that declines in phrenic motoneuron function are probably inevitable as the disease progresses (3, 4, 6, 13). Thus, respiratory failure will ultimately reflect impairments of the entire phrenic-diaphragm motor unit (i.e., motoneuron and myofibers).

The diaphragm is the primary breathing muscle and contraction enables tidal volume during resting breathing (21). When diaphragm integrity is critically compromised, life-saving correction is required to prevent death. Mechanical ventilation (MV) sustains ventilation in subjects with hypoventilation and respiratory failure and in many cases can stabilize patient-reported outcomes and maintain survival (22, 23). However, its use can also be associated with impaired quality of life (24, 25) and potential severe noninfectious (such as cardiovascular repercussion or barotrauma associated with high ventilator settings) and infectious (e.g., tracheitis, pneumonia) complications (26, 27). Additionally, prolonged use of MV and extended diaphragmatic inactivity may independently lead to severe diaphragm-muscle atrophy and dysfunction (ventilator-induced diaphragmatic dysfunction - VIDD), which may impede weaning from MV after an acute illness (28–30).

Diaphragm pacing (DP) is a form of respiratory support that generates ventilation via direct stimulation of the phrenic nerves to evoke diaphragm contraction. When used for patients with spinal cord injury (SCI), DP has been associated with reduced reliance on positive pressure MV (31–33). We previously reported an initial response to 6 months of DP, in three subjects with severe Pompe disease (34). This work demonstrated the feasibility of DP in this population, and provided initial evidence of a possible rehabilitative impact of DP. Further, the success of DP in that small sample of Pompe patients suggested that the diaphragm muscle was functional, but may not have been receiving optimal neural (phrenic) input during spontaneous breathing. The aim of this observational study was to build upon the prior report by comprehensively evaluating longer-term (e.g., up to 7 years post-DP implant) changes in respiratory function in adult and pediatric subjects with Pompe disease who underwent DP to treat ventilatory failure. We hypothesized that use of DP would preserve or improve the diaphragm activity and independent breathing ability of implanted patients.

## 2. Methods

### 2.1. Study design

This was a longitudinal observational study of changes in respiratory function and diaphragm activity following clinical implantation of DP. Study procedures were approved by the University of Florida Institutional Review Board, and the study was registered at www.clinicaltrials.gov (NCT02354651). The active study period ended in January 2019.

### 2.2. Participants

Subjects were eligible to participate if they were between ages 2 and 65 years; diagnosed with Pompe disease via mutational analysis or GAA enzyme activity assay in blood spot and/or fibroblast culture less than 40% of control values; exhibited chronic ventilatory insufficiency and functional quadriparesis; and were eligible to receive a diaphragmatic pacemaker as part of their clinical care. Subjects who required six or more daily hours of invasive (MV) or non-invasive ventilatory (NIV) support while awake and upright, for at least 21 days in duration, were considered to require prolonged mechanical ventilation (35).

Subjects, or their healthcare surrogate reviewed the informed consent form, received an opportunity to ask questions regarding the risks and benefits of their participation, and provided their consent to participate.

### 2.3. Diaphragm pacemaker

**Figure 1:**
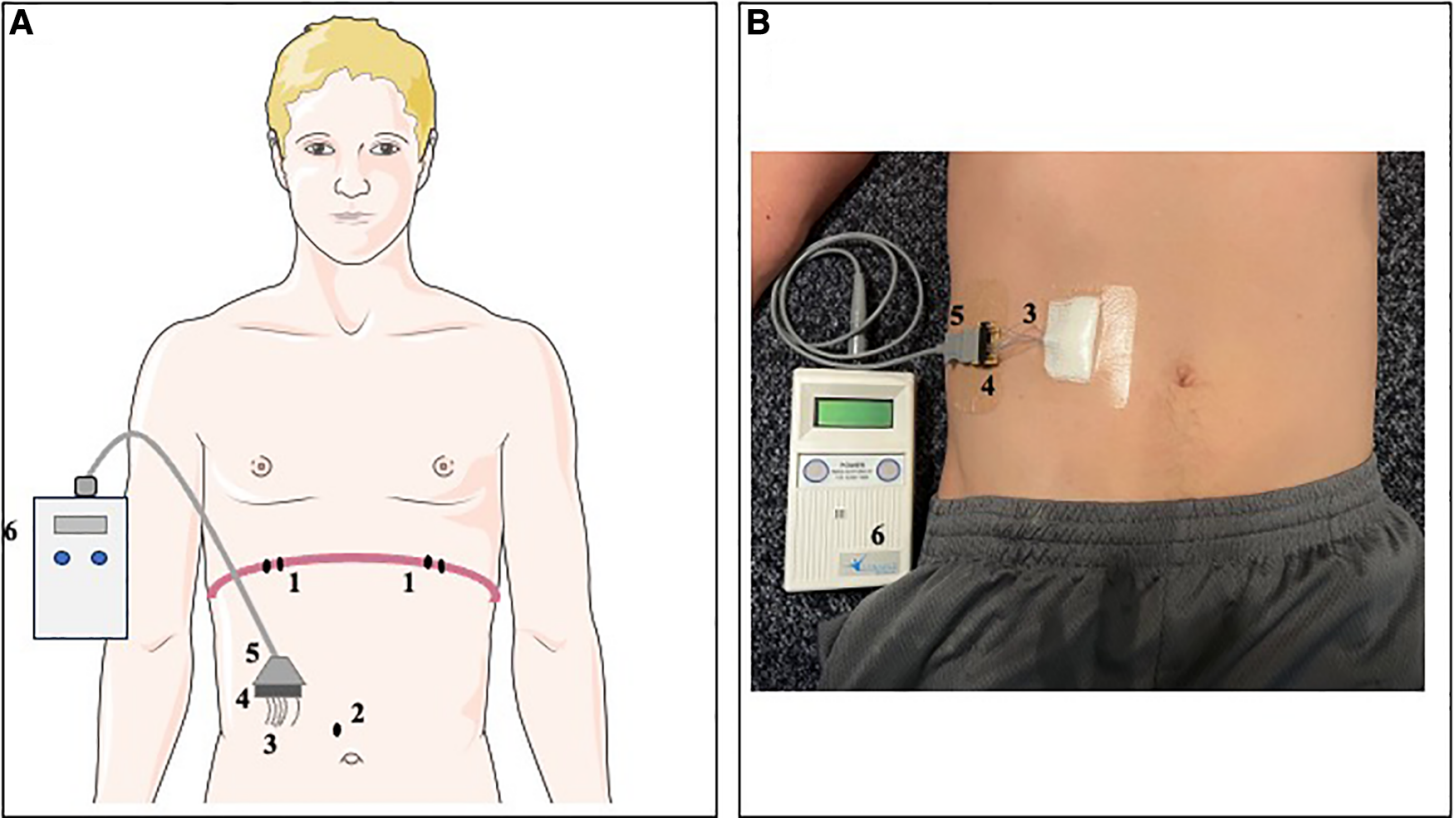
Representation of DP system in a schematic model (**A**) and simulated participant (**B**). Two bipolar electrodes (1) were laparoscopically implanted adjacent to the motor point of each hemidiaphragm. Additionally, a subcutaneous reference electrode (2) was implanted in the abdominal fascia. The four stimulation leads and reference electrode were externalized (3) in the right upper quadrant of the abdomen and mounted to an external connector block (4). An external connector cord (5) could be connected to the DP external pulse generator (6) to deliver stimulations at the programmed stimulation settings.

Local surgeons evaluated patients for eligibility for diaphragm pacing and implanted the NeuRx Diaphragm Pacing Stimulation system (DPS® NeuRx RA/4 system, Synapse Biomedical) as part of clinical management of chronic ventilatory insufficiency. Briefly, implantation occurred through laparoscopic mapping of diaphragm contractility to identify the motor point of each hemidiaphragm, followed by implantation of two bipolar stimulating electrodes in each hemidiaphragm motor point, plus a subcutaneous reference electrode (36). Lead wires were then externalized to a pacing socket and connected to a removable battery-operated external pulse generator (EPG). Figure 1 illustrates the components of the DP system. While the patient remained under anesthesia the surgical team determined the maximal stimulation amplitude and frequency that could be used. However, since sensory function remains intact with Pompe disease, the initial DP settings were determined only after the patient awakened. The stimulus parameters for each electrode were titrated to elicit a comfortable level of stimulation, and the respiratory rate was matched to the patient's spontaneous rate. The EPG was programmed to deliver a stimulation amplitude, frequency, and pulse width at a fixed respiratory rate, with the goal to deliver tidal volume and minute ventilation through the stimulated diaphragm contractions (37).

### 2.4. Diaphragm conditioning

Subjects began post-operative diaphragm conditioning as soon as possible (usually 3–5 days post-operatively) with an initial goal to tolerate 30 min of stimulation at the highest comfortable amplitude. The goal of diaphragm conditioning was to deliver a consistent stimulation that preserved inspiratory volume during reduced or removed positive pressure ventilation. Conditioning typically progressed by small increases in pacing duration every 2–5 days as tolerated, toward a goal of 24-hour pacing by day 30 post-surgery. The amplitude or pulse width of the external stimulator was increased on follow-up clinical visits, if the increase led to pain-free improvements in breath volume. In addition, subjects were encouraged to pace for short periods daily with reduced or no MV support. If subjects could not tolerate breathing without MV, they used the lowest MV support that yielded stable breathing for at least 20 min at a time.

### 2.5. Study measurements

Feasibility was measured through patient or parent reports of pacemaker use and tracking of adverse events. Response to pacing was evaluated by changes in positive pressure breathing support, breathing pattern without ventilator assistance, pulmonary functional tests, and diaphragm EMG. When possible, the respiratory and neuromuscular responses to diaphragm conditioning were measured within the first week, 1-month, and 6-month postoperative follow-ups, with longer-term repeated measurements every 1–2 years as able, when subjects were in their usual state of health. Test sessions occurred while patients were seated as upright as possible. Pulse oximetry, heart rate, respiratory rate, and end-tidal CO2 were monitored during test sessions.

#### 2.5.1. Breathing pattern

During each study visit, the tidal volume (VT), respiratory rate (RR) and minute ventilation (VE) were collected when possible. Airflow and pressure were sampled directly from the airway opening at 100 Hz frequency, using a pressure transducer and pneumotachograph connected to a clinical respiratory monitor (NM3, Philips-Respironics). Up to 20 min of ventilation were recorded both with and without the use of the diaphragm pacer, during the lowest tolerated support.

#### 2.5.2. Pulmonary functional tests

Pulmonary functional testing included peak cough flow and the pressure-generating capacity of the inspiratory muscles. Tests were administered in accordance with the American Thoracic Society and the European Respiratory Society guidelines (38).

Voluntary peak cough flow (PCF) represents the largest expiratory airflow recorded during a maximal volitional cough effort (39). PCF data were collected with a heated pneumotachograph (HR 3500B, 3700B, or 3813 Hans Rudolph, Shawnee, KS) connected to the tracheostomy or facemask. Since PCF can be influenced by inspiratory operating volume (40), patients above 4 years of age were instructed to take a deep breath and cough as forcefully as possible. Spontaneous coughs were recorded in preschool-aged children. Airflow from the pneumotach was recorded by a data acquisition system (S30-16 ADInstruments, Boston, MA) and laptop computer.

Maximal inspiratory pressure (MIP) represents the greatest pressure generated during a maximal voluntary inspiratory effort (41). A pressure transducer connected to a one-way valve was directly attached to the subject's tracheostomy (*n* = 5), or placed over the nose and mouth with a face mask (*n* = 1). A minimum of 3 trials was obtained; additional trials were administered if pressure efforts showed >20% variation. Participants were provided with as much rest as needed between efforts, and the average of the efforts was tracked over time.

#### 2.5.3. Diaphragm unstimulated EMG

Diaphragm activity was recorded during assessments of spontaneous (unpaced), off-ventilator minute ventilation and maximal inspiratory pressure. The external pacer leads were connected to a digital analog converter (PowerLab 16/30, ADInstruments) via a customized adaptor. Signals were sampled at 20 kHz, band-pass filtered (1–5,000 kHz).

#### 2.5.4. Data analysis

One-minute periods of breathing were selected for analysis. Selections were within the middle of the acquired breathing pattern sample and represented stable periods free from speaking, moving, or coughing. To evaluate longitudinal changes in the non-ventilated breathing pattern following DP, a mixed effects model with repeated measures was used. The independent variables were the time post-DP implant (4–6 weeks, 3–6 months, and 24 ± 3 months) and support (with or without DP). The time ranges were selected to maximize the number of available participants during each interval. For significant main effects, multiple comparisons were corrected using a Tukey test.

To prepare the diaphragm EMG signal for further analysis, ECG artifact was removed from the EMG signal, using an off-line gating approach. The EMG signal was rectified, and then high pass filtered (cutoff: 10 Hz) to remove T-wave offset. Then, the R wave was removed from the ECG channel by creating a short window (60–100 msec, depending on underlying heart rate), smoothing with a triangular window, and using the threshold feature to remove the portion of signal occurring during the QRS complex. To replace signal in areas where the QRS was removed, the EMG signal within the immediately preceding window was copied and inserted. The peaks of the root mean square of resting inspirations were averaged across one-minute intervals and normalized to the amplitude achieved during maximal inspiratory pressure. Using mixed effects models, two separate analyses evaluated changes in unpaced, off-ventilator spontaneous diaphragm activity over time; one analysis focused on changes in MIP-normalized EMG activity during resting breathing, and the second approach evaluated changes in EMG activity during resting breathing, using the coefficient of variation averaged across both hemidiaphragms. Sphericity was assessed with Geisser-Greenhouse's epsilon. For all statistical analyses, significance was *p* < 0.05.

## 3. Results

### 3.1. Participants

During the study period, nine subjects were enrolled. Two eligible patients elected not to undergo surgical placement, while a third participant was determined to be clinically ineligible for a DP implant due to coexisting medical conditions. Table 1 shows clinical characteristics of the six subjects who underwent DP. All required chronic, full-time MV at the time of implant (tracheostomy: *n* = 4, NIV: *n* = 2). Three subjects (one male, two females) were children diagnosed with EOPD in infancy who also required invasive feeding support, and the remaining three were adults with LOPD (two males, one female) who fed independently. Four subjects enrolled in the study upon DP implantation, while the other 2 subjects (subjects 4, 6) received DP at outside institutions and enrolled within 3 months following implantation. All subjects were evaluated at least 3 times, over a span ranging from early recovery up to 7 years post-DP.

**Table 1:** Sample demographics.

Subject ID	Sex	Age (years) at implant; (year of implant)	Support used at implant	Tracheostomy age (years)	Feeding tube	Maintenance ERT				Max off-vent tolerance pre-DP	Max off-vent tolerance post-DP
						Type	Duration	Dose	Frequency		
Subject 1	M	53; (2011)	MV	50	N	Lumizyme®	4 years	20 mg/kg	Biweekly	2 min	10 h. after 3 months.; ≥12 h. after 3 yrs.
Subject 2	F	3; (2012)	MV	3	Y	Lumizyme®	2.5 years	850 mg	Weekly	60 min	12 h. after 1 month.; ≥16 h. after 2 years.
Subject 3	M	48; (2013)	NIV	48	N	Lumizyme®	<0.5 years	1,400 mg	Biweekly	1 min (NIV)	1 h. after 3 months. (trach); 12 min. after 1 year.
Subject 4	M	4; (2015)	MV	3	Y	Myozyme®	3.5 years	40 mg/kg	Biweekly	0 min	0 min after 10 months.; 10 min after 29 months.
Subject 5	F	5; (2015)	MV	5	Y	Lumizyme®	4.5 years	500 mg	Biweekly	34 min	2 h. after 14 days; ≥12 h. after 4 months.
Subject 6	F	25; (2016)	NIV	N/A (NIV)	N	Lumizyme®	1 years	1,200 mg	Biweekly	1.5 min (NIV)	3 min. after 1 month.; 20 min after 15 months.; 50 min. after 26 months.

MV, mechanical ventilation; NIV, non-invasive ventilation.

### 3.2. Feasibility measurements

All subjects initiated clinical diaphragm conditioning within 5 days of implantation. Five subjects (83%) achieved 24-hours of DP use within the first month and thus commenced DP assisted breathing for short periods with reduced or no MV. Subject 1 was able to independently attach and detach the external pulse generator, but the remaining subjects required caregiver support to maintain a wearing schedule. Subject 6 tolerated pacing, but required physical assistance from outside caregivers to attach the external pulse generator. This subject commenced diaphragm conditioning with the pacemaker only when healthcare assistants visited the home (∼1–2 h/day) and did not consistently increase her DP use at long-term follow-up.

The use of the DP stimulator was periodically restricted by breaks in one or more external pacemaker leads. Five subjects (83%) experienced at least one lead break. However, 4 of the 9 lead breaks occurred in a single subject (subject 2). To reduce the likelihood of external lead damage, subject 2's diaphragm conditioning regimen was modified. The subject continued to breathe without support during waking hours, and DP stimulation was limited to periods when the subject was stationary in a wheelchair during school. Leads repairs were influenced by subjects' access to a trained DPS surgeon in their local area and occurred within a few days, up to several weeks following lead disruption. Following lead repairs, the ability to pace with reduced support resumed unimpeded. A lead break was considered rare for most individuals. On average, a lead break occurred every 3.7 person-years of DP during a cumulative 34 person-years of the study.

### 3.3. Adverse events

Every subject experienced one or more adverse events during the observational period, and two adult subjects were hospitalized for serious adverse events. Nearly all adverse events were medical complications of subjects' underlying severe Pompe disease and chronic ventilatory insufficiency. The only adverse events determined to be related to DP were the presence of skin irritation or scabbing of the external leads in 3 subjects (Table 2).

**Table 2:** Adverse events.

Subject ID	Adverse Event(s)	Serious	Related to Diaphragm Pacing	Outcome
1	Mucus plug (multiple)	Yes	No	Resolved
1	Irritation at leads (multiple)	No	Yes	Resolved
1	Pneumonia (multiple)	Yes	No	Resolved
2	Redness at leads (multiple)	No	Yes	Resolved
2	Spinal fusion surgery for scoliosis	Yes	No	Resolved
3	Tracheomalacia	Yes	No	Ongoing
3	Pneumonia (multiple)	Yes	No	Resolved
3	Status epilepticus and pneumonia	Yes	No	Resolved
3	Cholecystitis	No	No	Resolved
3	Femur fracture and status epilepticus	Yes	No	Resolved
4	Respiratory infection (multiple)	No	No	Resolved
4	Diarrhea (multiple)	No	No	Resolved
4	Occipital pressure ulcer	No	No	Resolved
5	Infusion port break	No	No	Resolved
5	Seasonal allergies/sinusitis	No	No	Ongoing
6	Irritation and scabs at leads (multiple)	No	Yes	Ongoing
6	Respiratory infection (multiple)	No	No	Resolved

### 3.4. Unassisted breathing

**Figure 2:**
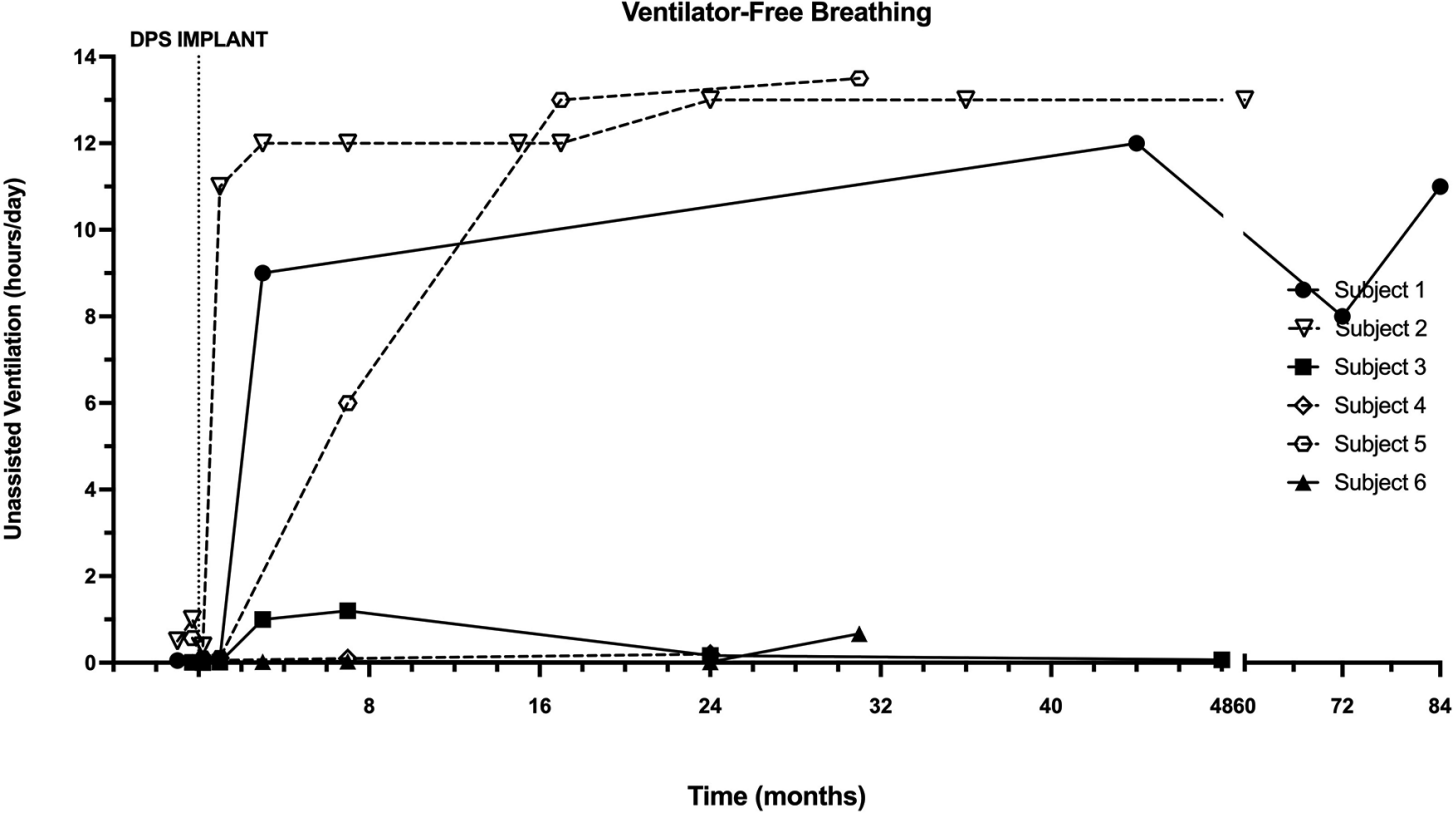
Longitudinal changes in ventilator-free breathing with DP. Ventilator-free breathing in each patient was recorded through interrogation of ventilator data chips during assessments following DP. One adult and two pediatric subjects achieved sustained weaning during waking hours, which typically occurred within the first 6 months.

Prior to DP implantation, all subjects required full-time positive pressure invasive or non-invasive ventilation (summarized in Table 1, individual case descriptions in Appendix). The daily assisted ventilation requirement generally decreased after diaphragm pacing implantation and conditioning (Figure 2). The individual subject responses to DP varied from a small improvement to a substantial reduction in the amount of daytime during which MV was required. Three subjects (subjects 1, 2 and 5) were fully weaned from daytime MV within 6 months of DP implantation. Longer-term follow-up revealed a largely preserved ventilator-free breathing while awake for up to 7 years post-implant.

#### 3.4.1. Effect of DP on unassisted breathing pattern

**Figure 3:**
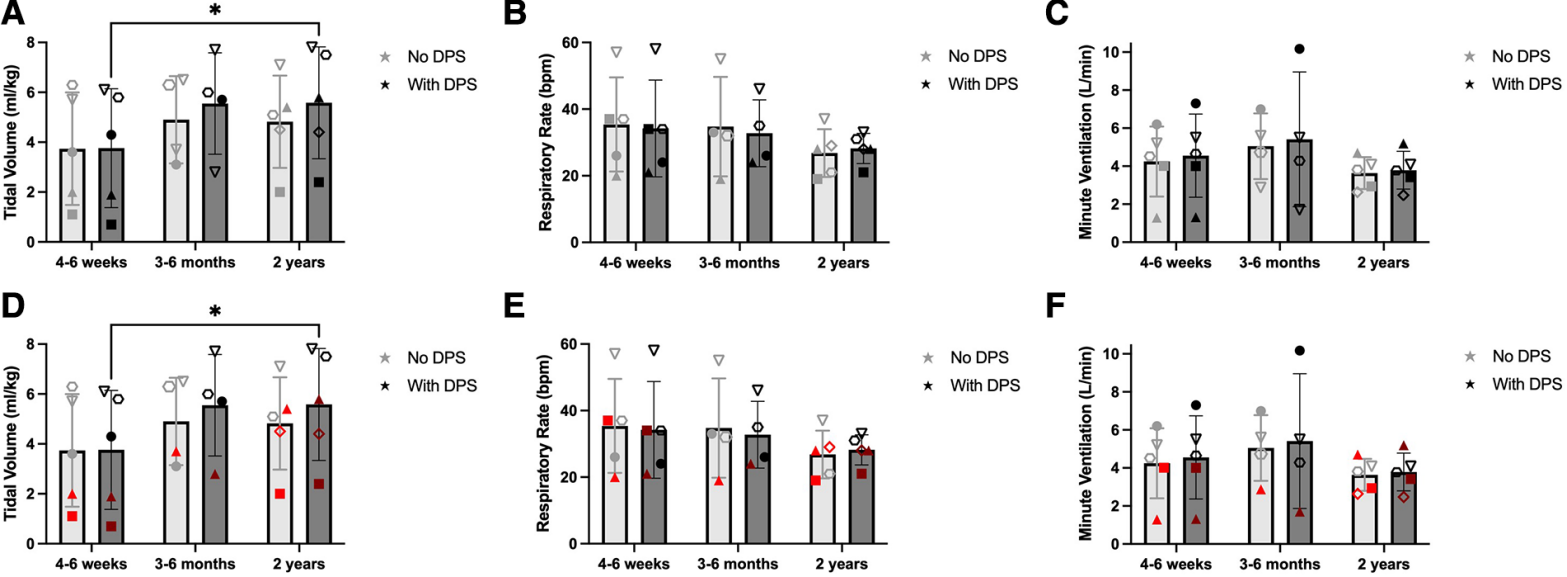
Effect of DP on unassisted breathing pattern. One-minute periods of off-ventilator breathing were with and without DP, and changes in ventilation were assessed between the first 4–6 weeks (*n* = 5), 3–6 months (*n* = 4), and 2 years (±3 months, *n* = 5) after DP. Paced tidal volume increased significantly between baseline and 2 years (**A**). Changes in respiratory rate (**B**) and minute ventilation (**C**) were more variable. Those who weaned during daytime hours (black) were observed to have greater tidal volume early after pacing (**D**) than those who continued full-time MV (red). Weaning-based differences in respiratory rate (**E**) or minute ventilation (**F**) were less discernable.

The timeframes for testing subjects varied due to their timing of enrollment, distance from our test center, ability to travel, and state of health. Thus, subjects were tested within the first 4–6 weeks (*n* = 5), 3–6 months (*n* = 4), and 24 ± 3 months (*n* = 5) following DP implant. Figure 3 illustrates differences in tidal volume, respiratory rate, and minute ventilation over the first two years post-DP. Values are compared for periods of no DP breathing vs. DP breathing. A significant time effect was detected for tidal volume (*p* = 0.031), with breath volume at 2 years significantly larger than 4–6 weeks. With No DP, tidal volume averaged: 4 wk: 3.7 ± 2.3 ml/kg, 6mo: 4.9 ± 1.8 ml/kg, 2y: 4.8 ± 1.8 ml/kg. Average VT values with DP were: 4 wk: 3.8 ± 2.4 ml/kg, 6mo: 5.6 ± 2.0 ml/kg, 2y: 5.6 ± 2.2 ml/kg. While DP appeared to increase average tidal volume at 6 and 24 months, these differences were not significant (*p* = 0.183). Significant differences in respiratory rate were not observed between on and off DP conditions (*p* = 0.657). This may be due to the fact that the rate of the DP stimulation was manually set to coincide with each subject's typical spontaneous breathing rate. Respiratory rate trended downward over time (*p* = 0.191). On average, the respiratory rate data for the No DP condition were: 4 wk: 35 ± 14 bpm, 6mo: 35 ± 15 bpm, and 2y: 27 ± 7 bpm. With DP, the values were: 4 wk: 34 ± 15 bpm, 6mo: 33 ± 10 bpm, 2y: 28 ± 5 bpm. Owing to the increased tidal volume, values for minute ventilation were elevated during DP, although this did not reach statistical significance due to extensive variability across subjects (*p* = 0.450). With No DP, minute ventilation values were: 4 wk: 4.2 ± 1.8 L/min, 6mo: 5.1 ± 1.7 L/min, 2y: 3.6 ± 0.8 L/min. With DP, minute ventilation values were 4 wk: 4.6 ± 2.2 L/min, 6mo: 5.4 ± 3.5 L/min, 2y: 3.8 ± 1.0 L/min.

**Figure 4:**
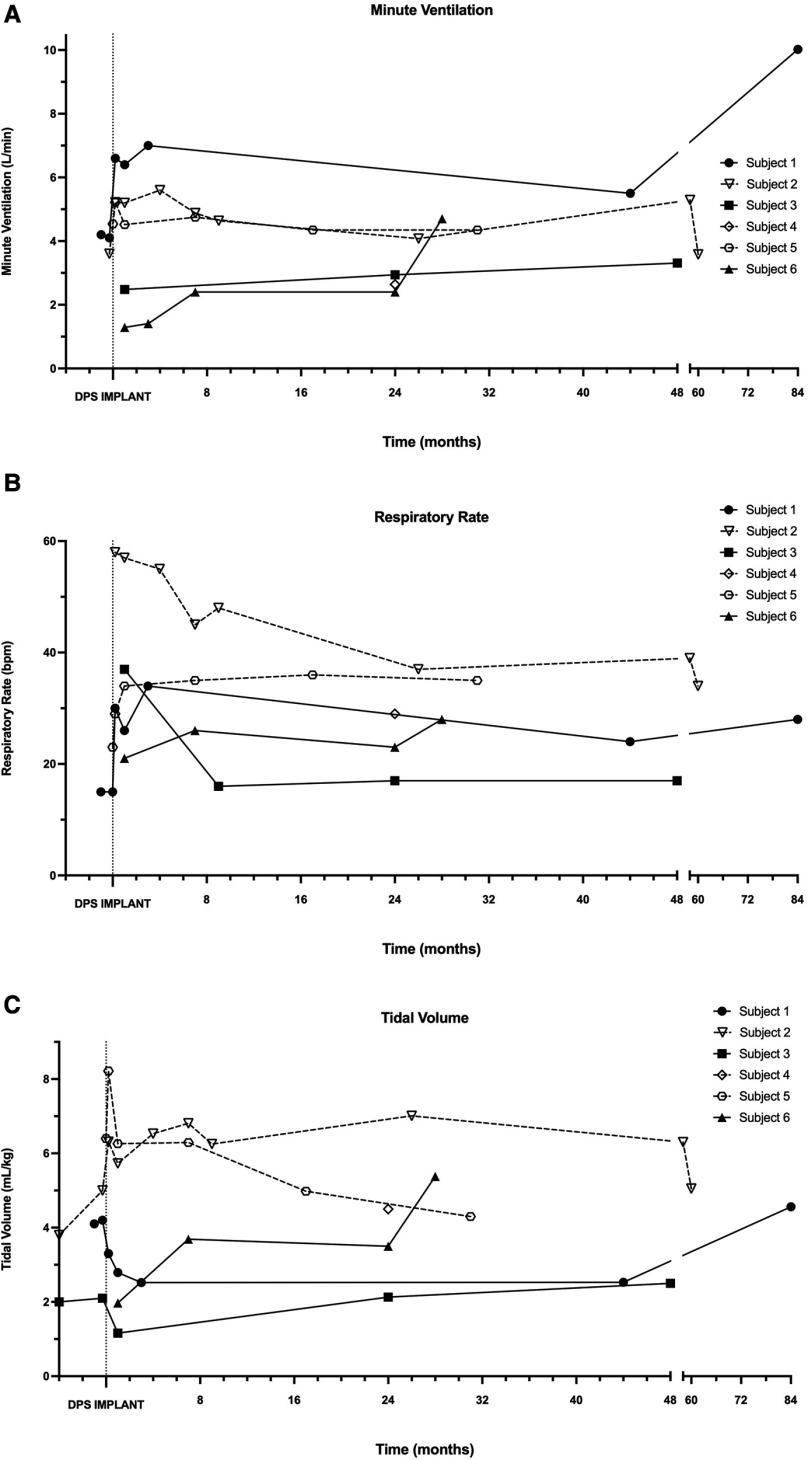
Longitudinal changes in independent minute ventilation, tidal volume and respiratory rate. During in-person assessments following DP implantation, minute ventilation (**A**), respiratory rate (**B**), and tidal volume (**C**) were recorded in each patient without the use of mechanical ventilation or DP.

Figure 4 depicts longer-term changes in the individual breathing pattern of each subject, without ventilator or pacer support. Most participants demonstrated a rapid and shallow breathing pattern immediately following DP implant, that stabilized within the first 3–6 postoperative months. While within-subject variability of breath volume and minute ventilation was noted, particularly for subjects 1, 2, and 3, most subjects' daily tolerance for off-ventilator breathing remained consistent over the duration of this observational study.

### 3.5. Pulmonary function

**Figure 5:**
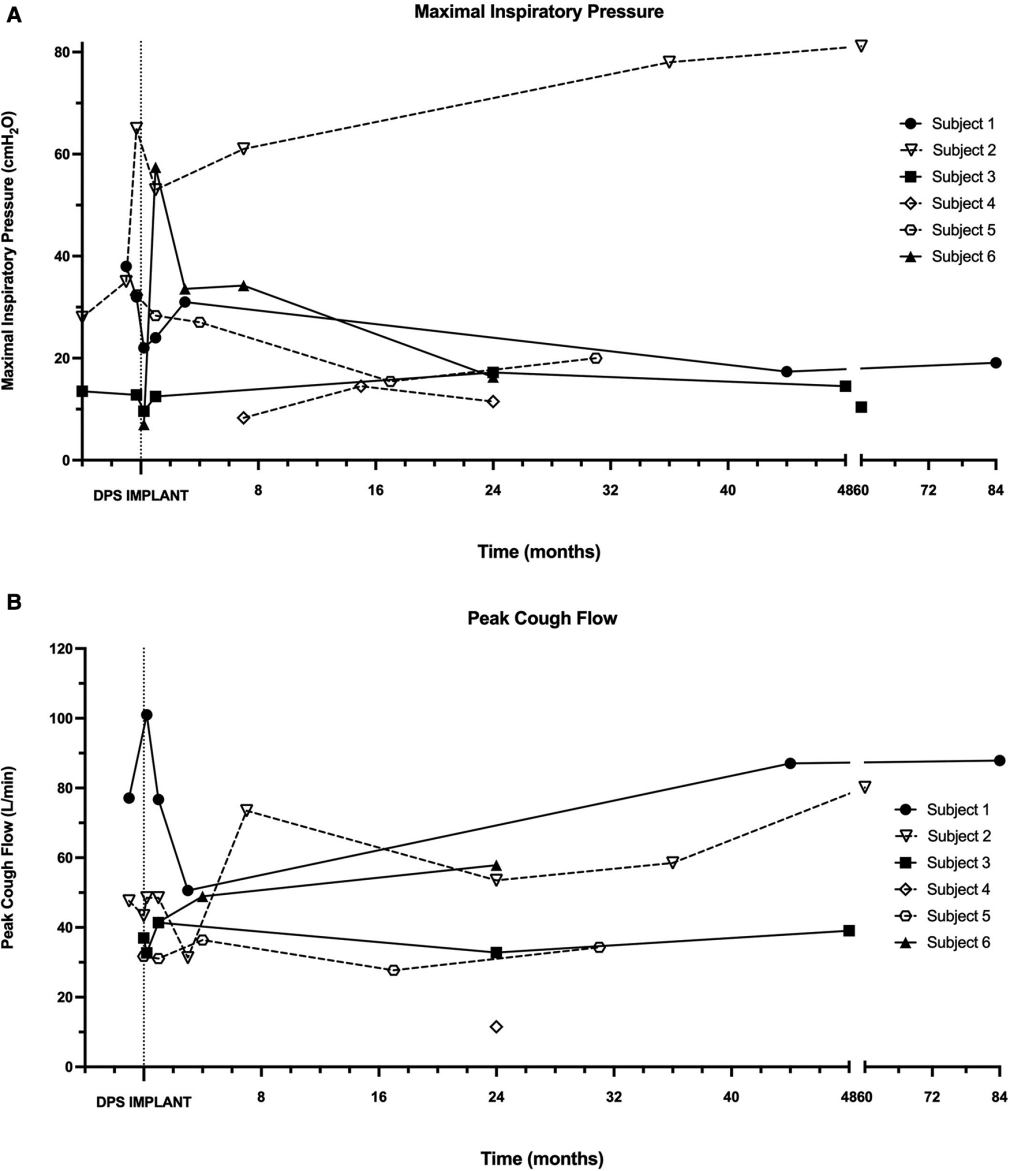
Maximal inspiratory pressure and peak cough flow. During in-person assessments following DP implantation, maximal inspiratory pressure (**A**) and peak cough flow (**B**) were recorded in each patient without the use of mechanical ventilation or DP.

Baseline pre-operative MIP and PCF were obtained from four subjects (subjects 1–3, 5). In each instance, MIP was profoundly impaired, and PCF values indicated an ineffective cough (Figure 5). Post-operatively, many subjects had modest decreases in MIP with a stabilization in PCF. One participant (subject 2) demonstrated large and sustained gains in both MIP and PCF post-implant.

### 3.6. Diaphragm electromyography

**Figure 6:**
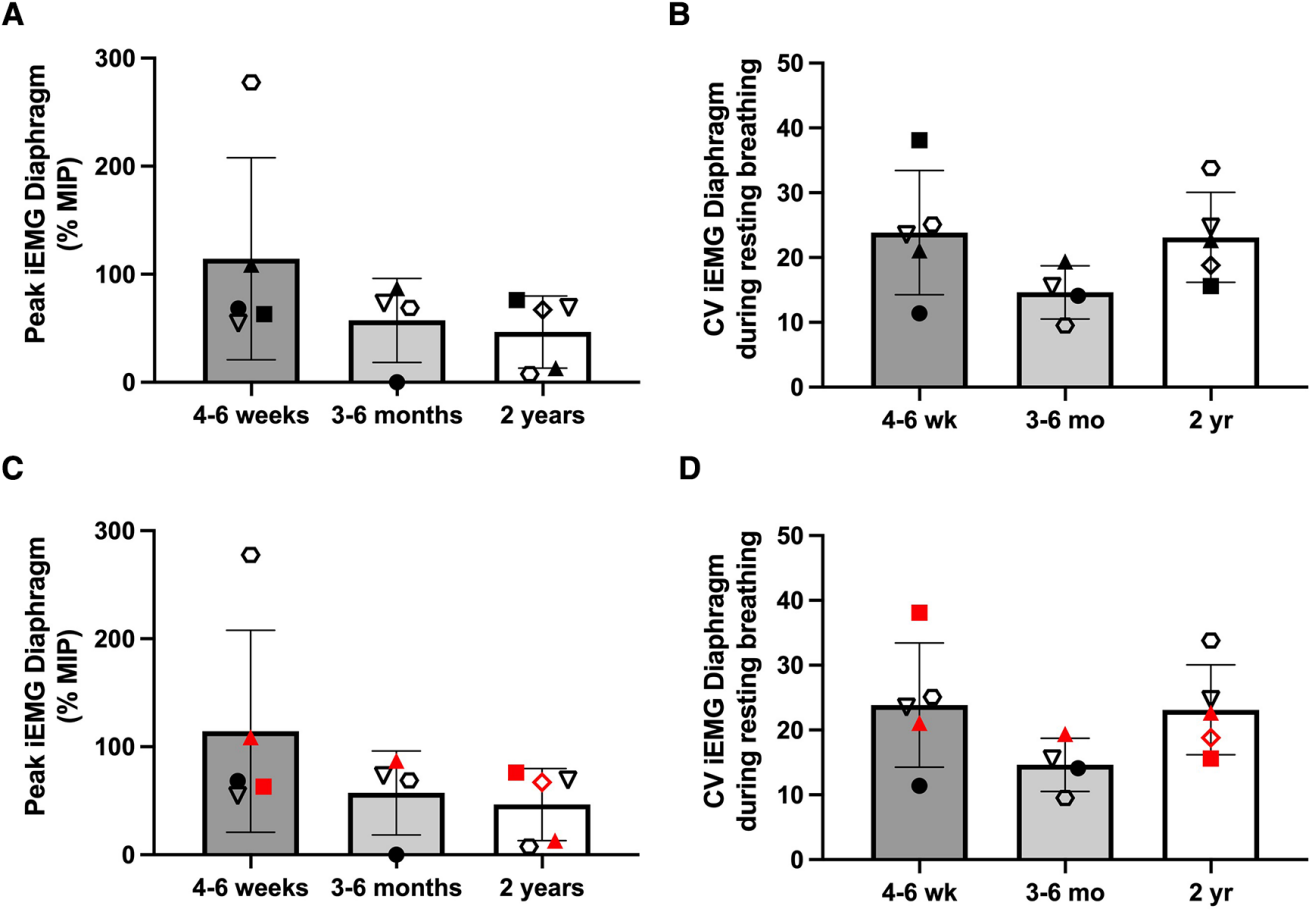
Longitudinal changes in diaphragm EMG during unassisted breathing. The peak integrated EMG of the right and left hemidiaphragms was recorded and averaged during off-ventilator, unpaced ventilation, and changes in activity were assessed between the first 4–6 weeks (*n* = 5), 3–6 months (*n* = 4), and 2 years (±3 months, *n* = 5) after DP. Individual results were variable, and no changes in peak iEMG (**A**) or coefficient of variation of the EMG (**B**) were detected. Further, no clear differences in peak iEMG (**C**) or coefficient of variation (**D**) were noted between those who weaned during daytime hours (black) and those who continued full-time MV (red).

**Figure 7:**
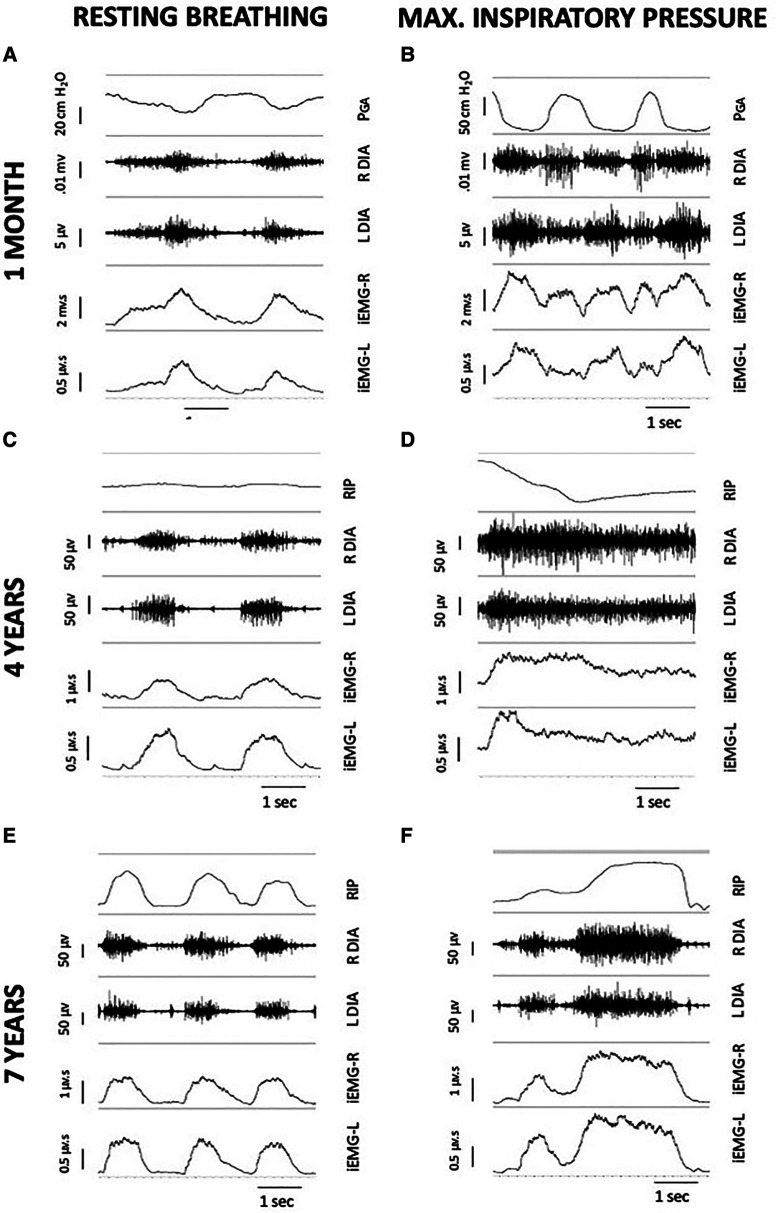
Serial, 5-second EMG recordings from indwelling right and left diaphragm electrodes of subject 1, during unpaced breathing without MV support (left column) and during MIP (right column). Each individual image includes a breath marker (either PGA or an abdominal RIP band), raw EMG of the right and left hemi-diaphragm, and a tracing of the integrated signal after cardiac filtering. At one month post-DP (**A,B**), PGA deflections remained negative during inspiratory efforts, indicating presence of a paradoxical breathing pattern and trunk rocking movements by the patient during off-ventilator breathing. At four years post-DP (**C,D**), the subject had repairs of DP leads and a ground wire replacement. Small, positive deflections of an abdominal RIP band during resting breathing (**C**) suggested emergence of diaphragm descent, with paradoxical movements during MIP due to predominant accessory muscle recruitment. At seven years post-DP (**E,F**), the ability to record inspiratory bursting from the diaphragm electrodes was retained in the subject, despite additional repairs of individual channel leads. DP: diaphragm pacing; EMG, electromyogram; MIP, maximal inspiratory pressure; MV, mechanical ventilation; PGA, gastric pressure; RIP, respiratory inductance plethysmography.

Inspiratory phasic bursting of the diaphragm was observed for all subjects while spontaneously breathing without MV or DP. Figure 6 depicts changes in the diaphragm peak integrated EMG (iEMG) amplitude and coefficient of variation of the iEMG, during unpaced, off-ventilator breathing, between 4 and 6 weeks (*n* = 5), 3–6 months (*n* = 4), and 2 years (±3 months, *n* = 5) following DP implant. The diaphragm EMG peak burst activity was normalized to the activation during the MIP maneuver (essentially, “% maximum”). Thus, a decline in normalized EMG output over time would indicate that spontaneous breathing was being achieved without needing to activate the diaphragm as intensely. This analyses suggested that diaphragm EMG activity trended downward over the duration of the study. However, between-subject variability was high, and no statistically significant changes were detected over time (*p* = 0.251). Variation in the subjects' breath-to-breath activation of the diaphragm was measured using the coefficient of variation of the iEMG signal and did not differ significantly (*p* = 0.185). While those who weaned from daytime MV tended to have greater iEMG coefficient of variation at 2 years than those who continued support, the low sample size did not permit us to test differences in activity between those who weaned from daytime MV and those who continued support. Notably, recordings from the indwelling electrodes yielded excellent EMG signal quality and enabled quantitative assessment of diaphragm activity of the subjects, for up to seven years following DP implant (Figure 7).

## 4. Discussion

This report outlines safety, feasibility, and longitudinal changes in breathing and diaphragm function following DP, in a small cohort of people with respiratory failure due to severe Pompe disease. It was noteworthy that following 2 years of DP use, an increase in unassisted tidal volume was detected in some subjects, accompanied by an increase in routine off-ventilator breathing time. This would not be expected to occur in the natural course of the disease. While the patient population remained prone to complications relating to their underlying neuromuscular disease, DP was well-tolerated. The few adverse events were related only to skin irritation and scabbing at points of wire externalization. Moreover, quantitative assessments of diaphragm activity during resting breathing and maximal voluntary efforts were a novel aspect of this study and possible for up to seven years, via recordings from indwelling electroides.

When unassisted breathing becomes impossible, one primary reason to consider the use of DP is that prolonged use of MV induces significant diaphragm atrophy and contractile dysfunction (28–30). The presence of ventilator-induced diaphragm dysfunction, or VIDD, is less understood in conditions where pre-existing neuromuscular dysfunction coincides with a new MV requirement (42). However, in a rat model, the presence of spinal cord injury greatly exacerbates VIDD (43). In the current cohort of Pompe patients, severe dysfunction of the phrenic motor system led to a chronic reliance on positive pressure ventilation. In some cases, the reliance of ventilator support was reduced for extended periods of the day following conditioning with DP. Thus, DP may be a supplemental approach that, when used in conjunction with other available strategies to preserve respiratory function, including enzyme replacement therapy, respiratory strength training, pulmonary hygiene, and respiratory support, may assist with stabilizing function. While many in the sample improved their ability to increase diaphragm activity and generate tidal volume over time, our methods do not permit us to directly attribute changes in independent breathing to an improved function of phrenic motor units.

The therapeutic benefits of phrenic stimulation to replace ventilation have been well-documented in those with SCI (44, 45). Use of DP early after SCI appears to increase the ability to voluntarily activate the diaphragm and generate tidal volume. Several groups have reported the use of DP to augment or completely replace positive pressure ventilation (33, 37, 46). Additionally, DP for SCI is associated with a reduced risk of respiratory infections (47) and improved quality of life (32). Beyond SCI, reports in congenital central hypoventilation syndrome (48), acute flaccid myelitis (49), and other neurological conditions (50) indicate off-label clinical use of DP can be implemented safely and associated with relatively few adverse effects (31).

Although cervical SCI disrupts the supraspinal control of the phrenic motor system, biology of the diaphragm remains relatively preserved. Thus, with sufficient phrenic stimulation, normal diaphragm contractility is the goal for DP in SCI (37, 44). When sensory loss accompanies SCI, patients can frequently tolerate high enough initial DP settings to enable a tidal volume sufficient for periods of off-ventilator, paced breathing (33, 51). It has been suggested that the DP settings can be increased upon implant in SCI, to the lowest settings necessary to achieve a functional stimulated breath volume (37, 51). Despite this guidance, the optimal DP “dose” and conditioning regimen for eliciting phrenic motor plasticity and ventilator weaning have not been evaluated in a controlled study.

In comparison to SCI, the pathophysiology of Pompe disease warrants a different approach – and expected clinical benefit – with DP. Sensory feedback remains intact in Pompe disease, and initial settings of the DP device were typically limited by participants' sensory tolerance. When subjects initiated diaphragm conditioning at our institution, we used a respiratory monitor to confirm a stable tidal volume and end-tidal CO_2_ during DP and recommended this approach whenever possible to avoid the presence of fatigue. Studies in an amyotrophic lateral sclerosis rodent model suggest that excessively high stimulation amplitude and frequency may contribute to motoneuron degeneration (52); thus, careful titration of stimulator amplitude and frequency is warranted. This initial descriptive study does not provide sufficient information to recommend an optimal “DP dose” to augment breathing ability in Pompe disease, but it does indicate that ongoing, Pompe related diaphragm or phrenic neuromuscular changes do not prevent DP from being effective.

Chronic ventilatory failure from Pompe disease accompanies fiber glycogenosis, muscle atrophy, and phrenic neuromuscular impairment (3, 4, 8). While DP could potentially counteract inactivity-induced weakness and atrophy of the diaphragm from prolonged MV in Pompe disease (4, 34), there is no evidence that DP would mitigate existing diaphragm pathology or reverse existing denervation of phrenic motor units. Moreover, coordination is required between the diaphragm and synergistic or accessory muscles of breathing and swallowing, to carry out complex respiratory-related behaviors and protective reflexes (53). Thus, normal minute ventilation and pulmonary function would not be a reasonable goal for DP in severe Pompe disease. On average, off-ventilator breath volumes of the sample (paced and unpaced) at 2 years post-DP increased by more than 20% over values early after implant. These relative gains in breath volume enabled daytime independence from MV in some subjects; those with low ventilation at the outset at implant remained largely MV-dependent. The small sample does not permit a detailed analysis of factors associated with daytime ventilator weaning. Moreover, since DP is not expected to alter the primary pathophysiology and clinical progression of Pompe disease, its therapeutic durability remains unknown.

Three subjects (subjects 1, 2, 5) achieved sustained daytime independence from MV with DP, which led to multiple quality of life enhancements. One subject was able to be discharged from a long-term facility to home, and another was able to be enrolled into regular classes at public school. Additionally, all three of these subjects were able to substantially reduce their requirements for home nursing and respiratory therapies. Overnight weaning from MV was not considered, due to the potential risk for central sleep apnea in Pompe disease (54). When DP was included in the acute care following spinal cord injury, it was associated with an average $144,444 in cost savings (55). The cost savings associated with use of DP to support daytime weaning are not understood for Pompe disease and outside the scope of this study.

The remaining subjects experienced an incomplete recovery of independent ventilatory function. Subject 3 was diagnosed with severe tracheomalacia and left mainstem bronchomalacia that required tracheostomy placement day 8 post-implant. Nevertheless, he was able to breathe up to 60 min without ventilator support at day 90, which had not been possible for at least 3 years prior to DP implant. The high-grade malacia necessitated some long-term positive pressure to prevent airway collapse, but over the following 3 years, his daytime inspiratory pressure requirement on the ventilator decreased from 29 cm H_2_O to <18 cm H_2_O. After 5 years of DP, the subject stopped DP and withdrew from participation, as his priorities shifted away from pacing. He passed away one year later. The major limitation for subject 4 was the rapid emergence of copious secretions without positive pressure ventilation. The patient did not have a functional swallow and required mechanical insufflation-exsufflation for airway clearance due to chronic aspiration. Despite the difficulty with secretion management, the subject was able to reduce MV inspiratory pressure from 26 cm H_2_O pre-implant to 22 cm H_2_O post-implant, and he improved from <1 min to 10 consecutive minutes of breathing using only DP. In the first post-operative year, subject 6 used DP for <2 h daily due to lack of available support at home. At 15 months post-implant, the subject was able to commit to 8 h of daily pacing, and daily off-ventilator time increased to 20 min. By the final visit, DP use increased to 24 h per day, and she reported 50 consecutive minutes of off-ventilator pacing. While these subjects did not experience sustained ventilator weaning, reported benefits of even short periods included an improved ability to smell, more natural speech, and greater ease with dependent transfers.

While DP appeared to stabilize or improve unassisted breathing over time, MIP continued to decline in most subjects. The diaphragm is a mixed muscle with approximately 50%–55% slow-twitch, fatigue resistance fibers, with the remaining fast-twitch fibers divided equally between fatigue-resistant and fatigable fibers (56). In healthy individuals, only a small percentage of the diaphragm's force generating capacity is needed for a tidal breath, presumably through contractions of slower, fatigue-resistant fibers. In contrast, robust diaphragm and inspiratory muscle recruitment occurs during MIP. Continuous, low-frequency chronic DP furthered fatigue resistance of diaphragm muscle fibers in human SCI (57). If similar fiber adaptations occurred with DP in Pompe disease, these fiber properties may have enabled longer independent ventilation at the expense of maximal foce generation during MIP or expulsive reflexes (58). Additionally, loss of pulmonary function is significantly associated with vastus lateralis muscle fiber damage in both infantile and older-onset Pompe phenotypes (59). In previous studies, vacuolated fibers and autophagic vacuoles were particularly observed in fast-twitch fibers of patients with Pompe disease and often preceded severe disease, along with a high occurrence of structural alterations in those with severe clinical disease (60). Observations from the diaphragm of one subject (subject 1) suggest that fiber degeneration observed in other patients with severe Pompe disease was not reversed by DP (4). The impacts of DP on an atrophic diaphragm require further clarification.

### 4.1. Limitations

This project studied an unusual clinical management approach for a rare disease. Thus, the small sample and heterogeneity of the participants limited our ability to perform detailed statistical analyses. While all participants exhibited functional dependence and chronic ventilatory failure, many had other medical comorbidities or underlying complications from long-standing Pompe disease (e.g., airway malacia, finger/hand contractures) that influenced their ability to utilize DP without interruption. Participants and their families shared anecdotal reports that some daily functional activities improved during DP, including improved trunk control and sitting endurance, breath and speech support, and airway clearance. However, we did not systematically administer a patient-reported outcome. A larger sample could help delineate the gains of DP as perceived by patients and their caregivers, and perhaps better distinguish the characteristics of DP responders vs. non-responders.

Indwelling EMG revealed robust inspiratory bursting during maximal inspiratory efforts and a potential trend toward lower relative recruitment of the diaphragm during tidal breathing. While this observation could be due to conditioning of the diaphragm, it is also possible that compensatory recruitment of the accessory inspiratory muscles contributed to a lower diaphragmatic recruitment during off-ventilator breathing. Moreover, compensatory abdominal muscle recruitment during expiration can also offset diaphragmatic weakness (61). Although the study design did not include diaphragmatic ultrasound, this approach could help distinguish contributions of the respiratory muscles during tidal breathing and maximal efforts. Our methods did not permit us to make discrete physiological comparisons between the diaphragm and accessory muscle activity, but the issue warrants further investigation.

While longitudinal assessments were conducted in the research lab whenever possible, most participants lacked the physical capacity to travel extended distances for follow-up. Moreover, some did not enroll into the study until after the DP implantation or were unavailable for regular home visits. These complications led to the inability to collect all measurements at least annually in every participant.

## 5. Conclusions

In conclusion, the results are consistent with our hypothesis that DP has the potential to augment ventilation in those with chronic ventilator failure due to Pompe disease. Recordings from long-term indwelling pacing electrodes provided further physiologic insights into diaphragm activity, for up to 7 years post-DP. In a multisystem, progressive condition such as Pompe disease, a multi-faceted approach is required to optimize function and improve the lived experiences of patients. DP may be an additional option beyond routine clinical care, particularly for those who are ineligible for other emerging treatments, such as next-generation enzyme replacement therapies or gene therapy. It is recommended that assessments of unassisted tidal volumes be included in preoperative evaluations of those under consideration for DP. While no firm conclusions can be drawn, diaphragm pacing may be considered as an adjunct in the treatment of this rare, debilitating condition.

## Data Availability

The raw data supporting the conclusions of this article will be made available by the authors, without undue reservation.
