# Supplementary material for: Diaphragm pacing and independent breathing in individuals with severe Pompe disease
Source: Front Rehabil Sci. 2023 Jul 31;4:1184031. doi: 10.3389/fresc.2023.1184031 (PMC10423945; doi:10.3389/fresc.2023.1184031)
Supplement: Supplementary file 1 [file Datasheet1.docx]

**Appendix 1.**

**Subject 1**

Subject 1 underwent DPS implant at age 53, following a 4-year history of chronic ventilatory failure and need for tracheostomy and invasive mechanical ventilation. Baseline comorbidities included recurrent atelectasis, respiratory infections, and mucus plugs. Prior to DPS implantation, the subject required 24-hour residential care and could only breathe independently for 2 minutes. Diaphragm conditioning started at post-op day #4. The subject tolerated 24-hour pacing within one month and was discharged to an acute rehabilitation hospital for one month of additional rehabilitation. By 3 months post-implantation, Subject 1 was able to spend up to 8 hours per day of paced breathing off the ventilator, and he was able to be discharged home with home health support. During our observations, the subject required one follow-up repair for breaks to 3 external leads, and he was hospitalized three different times for management of mucus plugs during periods of respiratory infection. At our final follow-up visit seven years after DPS implant, Subject 1 continued to use the pacer for 20 or more hours/day, and he spent up to 8 hours daily off the ventilator.

**Subject 2**

Subject 2 underwent DPS implantation at age 3, following a >2-year period of chronic ventilatory failure. Baseline comorbidities included dilated cardiomyopathy, cardiac hypertrophy, feeding difficulties requiring gastrostomy tube, chronic immunosuppression for ERT tolerance, and developmental delay. Subject 2 received a tracheostomy and mechanical ventilation two months prior to DPS implantation. As the lowered work of breathing alleviated some strain on the heart, the subject tolerated up to 1 hour of independent breathing at the time of surgery. Following surgery, diaphragm conditioning started at post-op day #4. Within three months, Subject 2 tolerated 24-hour pacing and up to 8 hours of breathing off the ventilator. With recovery of respiratory function, trunk control and antigravity upper extremity function also improved. The subject began scooting on the floor to move about the house and was able to propel the wheelchair. Two years following DPS implant, the subject underwent local Adeno-associated virus 1 (AAV1) mediated gene therapy to the diaphragm. Subject 2 experienced breaks of multiple external pacing leads in four different occasions; two of these required replacement of the ground lead by the surgeon. Coordination of DPS lead repairs led to a few interruptions of pacing for several weeks at a time, but in each instance, the subject could preserve independent daytime breathing. DPS use was then applied as 1-2-hour conditioning bouts to preserve diaphragm activation (e.g., more as a diaphragm exercise trainer than a breathing prosthesis). At our final follow-up visit six years after DPS implant, Subject 2 continued to use the pacer for 20 or more hours/day, and despite bilateral bronchomalacia, she remained off the ventilator up to 8 hours/day.

**Subject 3**

The subject underwent DPS implantation at age 48, following >5 years of chronic ventilatory failure. Baseline comorbidities included hypertension, hyperlipidemia, type II diabetes, history of pulmonary embolism, and stroke. Within the first postoperative week, severe tracheo-bronchomalacia was detected and managed with tracheostomy. Diaphragm conditioning started at post-op day #5, progressing to 24-hour pacing six weeks after implantation. Subject 3 was discharged to an acute rehabilitation hospital for approximately 5 weeks of additional rehabilitation, and then discharged to home with home health support. After a lead break during the acute hospitalization, no further lead breaks occurred for Subject 3. By 6 months post-implantation, Subject 3 was able to spend up to 1 hour per day of paced breathing off the ventilator. However, it became apparent that positive pressure would be needed to manage unresolved malacia of the large airways. DPS use continued, conditioning emphasized periods of lowered ventilator settings using reduced peak inspiratory pressure. After 2.5 years, DPS use was interrupted for ~3 months during recovery from seizures. At the final follow-up visit 4 years after implantation, the patient was able to spend up to 10 minutes of paced, off-ventilator breathing and several hours at reduced support. The patient experienced a femur fracture and additional seizures at 4.3 years post-implantation. After recovering from the hospitalization, the patient and his family elected to discontinue DPS use after 4.5 years. He passed away 6 months later following a deterioration in neurological status.

**Subject 4**

Subject 4 underwent DPS implantation at age 4, following a 1-year history of chronic ventilatory failure and tracheostomy placement for invasive ventilation. Baseline comorbidities included dilated cardiomyopathy from infancy, developmental delay, macroglossia, gastrostomy dependence, sialorrhea, multiple contractures, multiple episodes of respiratory failure, and pneumonia. Nocturnal ventilation was initiated at 2.5 years, and full-time invasive ventilation was required at age 3. An outside institution implanted a DPS and initiated conditioning within the first postoperative week, progressing to 24-hour pacing by the sixth postoperative week. The subject enrolled into the observational study two months after implant, and progressed from 1-2 minute tolerance of independent breathing preoperatively, to 10 minutes off-ventilator. Ventilator-free breathing remained limited by sialorrhea and excessive secretions, requiring frequent use of an insufflator-exsufflator device. Additionally, the subject experienced frequent respiratory infections each winter and interrupted pacing activities during these times. The subject was unable to pace for 2 months in 2017, following a lead break. At the time of study completion, Subject 4 used the pacer for 2.5 years and was able to spend 60-90 minutes using only continuous positive airway pressure, or up to 20 minutes of ventilator-free breathing.

**Subject 5**

The subject underwent DP implantation at age 5. The patient had tracheostomy placed for mechanival ventilation a following a 3-year history of chronic respiratory insufficiency, treated with overnight noninvasive ventilation. Eight months prior to DP implant, she experienced an aspiration event that led to acute ventilatory failure, tracheostomy, and inability to wean from mechanical ventilation. Other baseline comorbidities included dilated cardiomyopathy and cardiac hypertrophy, macroglossia, ggastrostomy dependence, multiple contractures, and scoliosis. The patient also had a remote history of AAV1 gene therapy to the diaphragm two years prior to the DPS implantation. The patient had a two-month pause of pacing in 2017, following a lead break. At the time of study completion, Subject 5 used the pacer for nearly 3 years and typically spent most daily waking, upright activities (~13.5 hours) ventilator-free using DP.

**Subject 6**

Subject 6 was diagnosed with Pompe disease at age 12 due to hypoventilation, failure to thrive, and loss of functional ambulation. Baseline comorbidities included scoliosis, spinal fusion at age 12, knee and hip flexion contractures, and a remote history of gastrostomy use . She required non-invasive ventilation for 11 years while supine and/or for sleeping (12-16 hours/day), and she reported difficulty feeding due to constant shortness of breath. Six months prior to DP implantation, she was hospitalized for a respiratory infection and required full-time non-invasive ventilation since. DP placement occurred at an outside institution, she initiated diaphragm conditioning the following day, and enrolled into the observational study eight days later. Due to weakness and contractures in the hands, the daily duration of diaphragm conditioning remained limited by the availability of outside caretakers, typically 1-2 hours/day. The subject was able to gradually increase conditioning in 2018, and at our final follow-up visit, Subject 6’s tolerance off-ventilator increased to 50-minute intervals with DP.
